# Dose–Response Matters! – A Perspective on the Exercise Prescription in Exercise–Cognition Research

**DOI:** 10.3389/fpsyg.2019.02338

**Published:** 2019-11-01

**Authors:** Fabian Herold, Patrick Müller, Thomas Gronwald, Notger G. Müller

**Affiliations:** ^1^Research Group Neuroprotection, German Center for Neurodegenerative Diseases (DZNE), Magdeburg, Germany; ^2^Department of Neurology, Medical Faculty, Otto von Guericke University, Magdeburg, Germany; ^3^Department Performance, Neuroscience, Therapy and Health, Medical School Hamburg, University of Applied Sciences and Medical University, Hamburg, Germany; ^4^Center for Behavioral Brain Sciences, Magdeburg, Germany

**Keywords:** physical activity, cognition, personalized training, personalized medicine, neuroplasticity, neuroprotection

## Abstract

In general, it is well recognized that both acute physical exercises and regular physical training influence brain plasticity and cognitive functions positively. However, growing evidence shows that the same physical exercises induce very heterogeneous outcomes across individuals. In an attempt to better understand this interindividual heterogeneity in response to acute and regular physical exercising, most research, so far, has focused on non-modifiable factors such as sex and different genotypes, while relatively little attention has been paid to exercise prescription as a modifiable factor. With an adapted exercise prescription, dosage can be made comparable across individuals, a procedure that is necessary to better understand the dose–response relationship in exercise–cognition research. This improved understanding of dose–response relationships could help to design more efficient physical training approaches against, for instance, cognitive decline.

## Introduction

In the last decades, the average time that people are physically active has decreased dramatically in Western countries (Owen et al., 2010; Church et al., 2011; Copeland et al., 2015), and physical inactivity has been named as a big, if not even the biggest, health problem of the twenty-first century (Blair, 2009). Remarkably, physical inactivity is associated with impaired cognitive functions (Aichberger et al., 2010; Falck et al., 2016; Ku et al., 2017; Tan et al., 2017) and higher risk of risk of neurodegenerative diseases (e.g., dementia) in the aging population (Laurin et al., 2001; Rovio et al., 2005; Ravaglia et al., 2008; Hamer and Chida, 2009; Paillard-Borg et al., 2009; Abe, 2012; Bowen, 2012; de Bruijn et al., 2013; Grande et al., 2014; Paillard, 2015). In order to counteract such negative effects of physical inactivity, an increase in the habitual physical activity level, which is typically engendered through a regular engagement in physical exercises, is empathically recommended (Hillman et al., 2008; Bherer et al., 2013; Erickson et al., 2013, 2014; Hötting and Röder, 2013; Paillard, 2015; Müller et al., 2017; Liu-Ambrose et al., 2018; Herold et al., 2019). It has been well demonstrated in the literature that a single bout of physical exercises (Chang et al., 2012a; Basso and Suzuki, 2017; Herold et al., 2018b; McSween et al., 2018; Moreau and Chou, 2019) as well as repeated sessions of physical exercises in the form of a training intervention (e.g., endurance training) (Colcombe et al., 2006; Erickson et al., 2011; Voelcker-Rehage et al., 2011; Herold et al., 2018b, 2019; Falck et al., 2019; Stern et al., 2019) induce substantial neurocognitive changes. Based on such positive effects of physical exercises and/or physical training on brain plasticity and on cognition, it is not surprising that many scientific disciplines (e.g., medicine, psychology, neuroscience, and sport science) pay attention to this research field. Although these different scientific disciplines use different approaches to understand the relationship between physical exercises and/or physical training and the central nervous system, it is undoubted that all of them are based on an appropriate exercise prescription that specifies exercise (e.g., exercise intensity, exercise duration) and/or training variables (e.g., frequency of training sessions) (Lightfoot, 2008; Williams et al., 2019). Furthermore, exercise prescription is the key for dosing (Wasfy and Baggish, 2016; Pontifex et al., 2018) and for individualization of acute physical exercises and physical training (Lightfoot, 2008). Individualization (personalizing) is an emerging approach aiming at maximizing the efficiency of an intervention by accounting for the interindividual heterogeneity in the response to acute physical exercises and/or physical training (Lightfoot, 2008; Buford et al., 2013; Barha et al., 2017b; Müller et al., 2017, 2018; Gallen and D’Esposito, 2019). Notably, what parameters are optimal to prescribe the best exercise for an individual is extensively discussed in the literature (Katch et al., 1978; Weltman et al., 1989, 1990; Gass et al., 1991; Meyer et al., 1999; Hofmann and Tschakert, 2010; Scharhag-Rosenberger et al., 2010; Mann et al., 2013; Weatherwax et al., 2016), but not all scientific disciplines investigating exercise–cognition are taking this issue into account sufficiently (Gronwald et al., 2018b, 2019a; Suwabe et al., 2018). Hence, the purpose of this article is to shed light on differences in exercise prescription and their relation to the dose and the interindividual heterogeneity in neurocognitive outcome measures.

### Physical Activity, Physical Exercises, Physical Training – Where Are the Differences?

Prior to going more deeply into the topic of physical activity, physical exercise, and/or physical training, it is necessary to clarify these terms because they represent different concepts while it is, unfortunately, common behavior to use them interchangeably (Caspersen et al., 1985; Budde et al., 2016). “Physical activity” is defined as muscle-induced bodily movement that increases energy expenditure above ∼1.0/1.5 MET (metabolic equivalent of task; 1 MET = 1 kcal (4,184 kJ) × kg^–1^ × h^–1^) (Caspersen et al., 1985; Ainsworth et al., 2000; Budde et al., 2016). Hence, the term physical activity is a hypernym (i) that covers a wide range of physical activities that are conducted on a regular or unregular basis in a relatively unstructured and unplanned manner and (ii) that includes specific, planned, and structured forms of physical activities that are known as physical exercises (Caspersen et al., 1985; Howley, 2001; Budde et al., 2016). Physical exercises should be distinguished based on temporal characteristics into acute physical exercise (single bout) and chronic physical exercises (repeated bouts of acute exercises) (Scheuer and Tipton, 1977; Budde et al., 2016). A single bout of physical exercise is commonly referred to as an “acute (single) bout of physical exercise” or as “acute physical exercises” (Budde et al., 2016; Herold et al., 2018b). Furthermore, chronic physical exercises can be denoted as “physical training” when they are conducted regularly in a planned, structured, and purposive manner with the objective to increase (or maintain) individual capabilities in one or multiple fitness dimensions (Scheuer and Tipton, 1977; Caspersen et al., 1985; Howley, 2001; Budde et al., 2016; Herold et al., 2018a). In essence, distinguishing and using these terms carefully allows a better classification and interpretation of observed effects and of the underlying (neuro)biological mechanisms (Budde et al., 2016).

### “Responder” or “Non-responder” – That Is the Question

Since every human is unique, there is a considerable amount of within-individual (intra-individual) (Katch et al., 1982; Coggan and Costill, 1984; Bagger et al., 2003; Skurvydas et al., 2010; Faude et al., 2017; Chrzanowski-Smith et al., 2019; Voisin et al., 2019) and between-individual (interindividual) heterogeneity (Karavirta et al., 2011; Chmelo et al., 2015; Bonafiglia et al., 2016; Greenham et al., 2018) in acute psychophysiological response(s) to the same acute physical exercises and/or long-term adaptions to the same physical training. Especially, the interindividual heterogeneity gained attention in the research of the recent years (Buford and Pahor, 2012; Buford et al., 2013; Mann et al., 2014; Sparks, 2017; Pickering and Kiely, 2018b; Ross et al., 2019) and is commonly observed in studies dealing with endurance (cardiovascular) training (Chmelo et al., 2015; Bonafiglia et al., 2016), resistance (strength) training (Hubal et al., 2005; Chmelo et al., 2015; Ahtiainen et al., 2016), or combined training (consisting of endurance and resistance training) (Karavirta et al., 2011). In order to account for this interindividual heterogeneity, the concept of (i) “responder” [also referred as “individuals with high sensitivity” (Booth and Laye, 2010)] and (ii) “non-responder” [also referred as “individuals with low-sensitivity” (Booth and Laye, 2010), limited responders (Burley et al., 2018), or “individuals which did not respond” (Pickering and Kiely, 2018b)] was introduced, however, with varying definitions (Booth and Laye, 2010; Buford and Pahor, 2012; Scharhag-Rosenberger et al., 2012; Buford et al., 2013; Mann et al., 2014). While the definition and methods to classify responders and non-responders are currently discussed in the literature (Atkinson and Batterham, 2015; Hecksteden et al., 2015, 2018; Bonafiglia et al., 2018, 2019a,b; Swinton et al., 2018; Atkinson et al., 2019; Dankel and Loenneke, 2019), it is relatively accepted that (i) not all outcome variables are affected equally by the responsiveness state (e.g., be a responder or non-responder) (Sparks, 2017; Pickering and Kiely, 2018b, 2019b; Toigo, 2019), (ii) measurement errors are inevitable in repeated measurements and are caused, for instance, by random biological fluctuations that do not represent a meaningful change in the outcome variable (Atkinson and Nevill, 1998; Scharhag-Rosenberger et al., 2012; Atkinson and Batterham, 2015; Williamson et al., 2017; Pickering and Kiely, 2019a), and (iii) some responses are likely to be transient, causing uncertainty regarding the time course of the responsiveness state (Pickering and Kiely, 2018b). Hence, the following working definitions can be proposed (see Table 1). Regarding the response to acute physical exercises and/or physical training, (i) responders are individuals who exhibit, at a certain time point, changes in a variable of interest that are above (below) a distinct threshold, and (ii) non-responders are individuals who exhibit, at a certain time point, changes in a variable of interest that are below (above) a distinct threshold. There is ongoing vivid discussion on how to define these critical thresholds (Atkinson and Batterham, 2015; Swinton et al., 2018; Atkinson et al., 2019; Dankel and Loenneke, 2019) and whether further subgroups should be established (Dankel and Loenneke, 2019). For instance, “adverse responders” (Bouchard et al., 2012) or “negative responders” (Leifer et al., 2014), have be defined as individuals who exhibit, at a certain time point, in response to acute physical exercise or physical training, unfavorable responses below (above) a distinct threshold. In addition, “above” and “below” need to be referenced relative to a specific outcome in the variable of interest. For instance, in a cognitive test, performance could be operationalized by “number of correct items” and “reaction time” (variables of interest). Regarding number of correct items, it is favorable to achieve a higher number of correct items (responder: above; non-responder: below). Regarding reaction time, on the other hand, it is favorable to react faster (responder: below; non-responder: above). Regardless of the ongoing discussion about how to classify the level of responsiveness, there is some evidence that the interindividual heterogeneity in response to acute physical exercise and/or physical training might contribute to the interindividual heterogeneity observed in neurocognitive outcomes. This evidence is outlined in the following section.

**TABLE 1:** Overview about the definitions of terms relevant to interindividual heterogeneity and exercise–cognition research ^∗^ Please note that “above” and “below” are relative to the favorable outcome in the variable of interest.

**Definition of terms relevant to interindividual heterogeneity and exercise–cognition research**	
Physical activity	“Physical activity” is any muscle-induced bodily movement that increases energy expenditure above ∼1.0/1.5 MET (metabolic equivalent of task; 1 MET = 1 kcal (4,184 kJ) × kg^–1^ × h^–1^) (Caspersen et al., 1985; Ainsworth et al., 2000; Budde et al., 2016).
Physical exercise	“Physical exercises” are specific, planned, and structured forms of physical activities (Caspersen et al., 1985; Howley, 2001; Budde et al., 2016) and should be distinguished on temporal characteristics into (i) *acute physical exercise* (single bout) and *chronic physical exercises* (repeated bouts of acute exercises) (Scheuer and Tipton, 1977; Budde et al., 2016).
Physical training	“Physical training” is chronic physical exercises when they are conducted regularly in a planned, structured, and purposive manner with the objective to increase (or maintain) individual capabilities in one or multiple fitness dimensions (Scheuer and Tipton, 1977; Caspersen et al., 1985; Howley, 2001; Budde et al., 2016; Herold et al., 2018a).
External load	“External load” along with influencing factors (e.g., climatic conditions, equipment, ground condition) is defined as the work completed by the individual independent of internal characteristics (Wallace et al., 2009; Halson, 2014; Bourdon et al., 2017; Burgess, 2017; Vanrenterghem et al., 2017; McLaren et al., 2018; Impellizzeri et al., 2019).
Internal load	“Internal load” is defined as individual and acute biomechanical, physiological, and/or psychological response(s) to the influencing factors (e.g., climatic conditions, equipment, ground condition) and the work performed (external load) (Wallace et al., 2009; Halson, 2014; Bourdon et al., 2017; Burgess, 2017; Vanrenterghem et al., 2017; McLaren et al., 2018; Impellizzeri et al., 2019).
Dose	“Dose” is commonly defined as a product of exercise variables (e.g., exercise intensity, exercise duration, type of exercise), training variables (e.g., frequency of training sessions), and the application of training principles (Wasfy and Baggish, 2016; Northey et al., 2017; Solomon, 2018; Cabral et al., 2019; Erickson et al., 2019; Etnier et al., 2019; Falck et al., 2019; Ross et al., 2019; Williams et al., 2019) and should be operationalized by using a specific marker(s) of internal load. The specific marker(s) should be involved in biological processes driving the desired changes (e.g., lactate → brain-derived neurotrophic factor (BDNF) → neurocognitive changes).
Responder	“Responders” are individuals who exhibit, at a certain time point, changes in a variable of interest that are above (below^∗^) a distinct threshold.
Non-responder	“Non-responders” (or “individuals which did not respond”) are individuals who exhibit, at a certain time point, changes in a variable of interest that are below (above^∗^) a distinct threshold.

**For instance, in a cognitive test performance, variables of interest could be operationalized by “number of correct items” and “reaction time.” In “number of correct items,” it is favorable to achieve a higher number of correct items. Hence, “responders” are individuals who exhibit at a certain time point changes in a variable of interest that are above a distinct threshold, and “non-responders” are individuals who exhibit, at a certain time point, changes in a variable of interest that are below a distinct threshold. In contrast, regarding reaction time, it is favorable to react faster. Hence, “responders” in this context are individuals who exhibit, at a certain time point, changes in a variable of interest that are below a distinct threshold, and “non-responders” are individuals who exhibit at a certain time point changes in a variable of interest that are above a distinct threshold.**

### Responsiveness State, Functional and Structural Brain Changes, and Cognition

In the following, we will refer to acute endurance exercises and endurance training because (i) from a neuroevolutionary view, endurance capacities (e.g., running during foraging) are important to ensure physical and/or neurocognitive well-functioning (Mattson, 2012; Raichlen and Alexander, 2017), (ii) acute endurance exercises and/or endurance training are currently in the focus of exercise–cognition research (Hillman et al., 2008; Stimpson et al., 2018), (iii) endurance training induces substantial structural brain changes (Erickson et al., 2011; Stern et al., 2019), and (iv) endurance training entails greater benefits in cognitive performance than resistance training (Barha et al., 2017a).

#### Acute Physical Exercises

With regard to acute physical exercises, it was observed that individual baseline working memory function was linked with changes in working memory performance following acute very-light-to-moderate-intensity endurance exercises (Yamazaki et al., 2018). Furthermore, responders, who showed improved cognitive performance after a single bout of very-light-to-moderate-intensity endurance exercise, exhibited a higher level of prefrontal activation during exercising (Yamazaki et al., 2017). This finding suggests that not only peripheral systems are affected by the individual responsiveness state but also the central nervous system itself.

#### Physical Training

Changes in cardiorespiratory fitness (CRF) measures in response to a 20 weeks endurance training program tended to vary tremendously among individuals (Bouchard et al., 1999). Exemplarily, Karavirta et al. (2011) observed, after a 21 weeks long combined exercise intervention in older adults, changes in CRF levels [assessed via maximal oxygen uptake (VO2 max.)] ranging from -8 to 42%. Such interindividual differences in response to endurance training also affect the expression of neurotrophic factors [e.g., brain-derived neurotrophic factor (BDNF)] (Heisz et al., 2017), which play an important role in neuroplasticity (Erickson et al., 2010, 2011; Brigadski and Leßmann, 2014) and cognition (Erickson et al., 2010; Brigadski and Leßmann, 2014; Leckie et al., 2014). For instance, after 6 weeks of high-intensity endurance training, responders who improved their CRF [assessed via peak oxygen uptake (VO2 peak)] to a greater extent than non-responders exhibited a significantly higher increase in serum BDNF (Heisz et al., 2017). This finding suggests that the state of responsiveness influences important mechanisms involved in neuroplasticity and cognition. Notably, a higher level of CRF level [mostly operationalized by VO2 max. (highest value attainable by a subject) or VO2 peak (highest, “system-limited” value attained during the CRF test) (Day et al., 2003)] is associated with (i) better cognitive performance in younger adults (Suwabe et al., 2017; Wengaard et al., 2017; Fortune et al., 2019), older adults (Erickson et al., 2011; Bugg et al., 2012; Verstynen et al., 2012; Albinet et al., 2014; Freudenberger et al., 2016; Hayes et al., 2016; Bherer et al., 2019; Castalanelli et al., 2019; Pentikäinen et al., 2019), and older adults with mild cognitive impairments (Sobol et al., 2018); (ii) favorable functional brain changes in younger adults (Stillman et al., 2018) and older adults (Colcombe et al., 2004; Albinet et al., 2014; Dupuy et al., 2015; Hyodo et al., 2016); and (iii) favorable structural brain changes in older adults (Colcombe et al., 2003; Erickson et al., 2009; Szabo et al., 2011; Bugg et al., 2012) and individuals with Alzheimer’s disease (Burns et al., 2008; Honea et al., 2009; Vidoni et al., 2012). Furthermore, in response to endurance training, increases in VO2 max. (iv) mediate the improvement in cognitive functions in younger adults (Stern et al., 2019) and (v) are associated with increases in hippocampal volumes in older adults (Erickson et al., 2011).

In sum, based on these associations between measures of CRF, measures of brain function and structure, and cognitive performance measures (albeit these correlations are not strictly causal in nature), it seems plausible to hypothesize that the large interindividual heterogeneity in measures of CRF may also contribute, among other factors, to the interindividual heterogeneity in measures of neurocognition in response to endurance training. However, to clarify the validity of these assumptions, further research is required.

### Master (of) the Fate? – How Genetics and Lifestyle Contribute to Interindividual Heterogeneity

The interindividual responsiveness to physical exercises and/or physical training and, in turn, the interindividual heterogeneity in outcomes are caused by several moderators, including both non-modifiable factors (e.g., sex or genotypes) and modifiable factors (e.g., nutrition, social or cognitive activities, exercise prescription) (Spiering et al., 2008; Bamman et al., 2014; Mann et al., 2014; Erickson et al., 2015; Sparks, 2017; Pickering and Kiely, 2018b). Notably, these factors can also be classified as endogenous factors (factors attributable to the individual such as sex or genotypes) and exogenous factors (factors attributable to external inputs, e.g., generated by exercise prescription) (Sparks, 2017). Currently, the roles of non-modifiable (endogenous) factors such as sex (Barha et al., 2017a, b, 2019; Barha and Liu-Ambrose, 2018; Cobbold, 2018; Loprinzi and Frith, 2018; Dao et al., 2019) and genotypes (Booth and Laye, 2010; Timmons et al., 2010; Timmons, 2011; Bouchard, 2012, 2019; Mann et al., 2014; Bouchard et al., 2015; Jones et al., 2016; Pickering and Kiely, 2017a,b,c, 2018a; Del Coso et al., 2018) are investigated most. Among these factors, it has been shown that a considerable amount (approximately up to half of the variance) of the interindividual heterogeneity in physical outcomes (Bouchard and Rankinen, 2001; Timmons et al., 2010; Davidsen et al., 2011; Timmons, 2011; Bouchard, 2012; Wilson et al., 2019), cognitive outcomes (McClearn, 1997; Goldberg and Weinberger, 2004; Blokland et al., 2008; Erickson et al., 2008; Friedman et al., 2008; Canivet et al., 2015, 2017), and brain structure outcomes (Thompson et al., 2001; Toga and Thompson, 2005; Bueller et al., 2006) are explained by genetics. However, considering current evidence, lifestyle factors may equalize a “genetic handicap” since people with a high CRF level but “unfavorable” genetic polymorphisms do not need to perform significantly poorer than individuals with low CRF level but “favorable” genetic equipment (Brown et al., 2019). These findings suggest that a genetic handicap can be counteracted by other factors (Flück, 2018) and that “overemphasizing” genetics for the individualization of exercise prescriptions is counter-productive (Carlsten and Burke, 2006; Kohane, 2009; Horwitz et al., 2013; Joyner and Lundby, 2018; Peck, 2018; Joyner, 2019). However, analysis of the genetics of participants is undoubtedly helpful in supporting individualization of acute physical exercise and/or physical training by aiding, for instance, the identification of potential responders and non-responders (Lightfoot, 2008; Pescatello, 2008; Booth and Laye, 2010; Timmons et al., 2010; Timmons, 2011; Pickering and Kiely, 2019a, b). Remarkably, it has also been highlighted that no “global non-responders” exist (Ross et al., 2015; Bonafiglia et al., 2016; Montero and Lundby, 2017; Pickering and Kiely, 2017c, 2018b; Toigo, 2019). Moreover, it is assumed that non-responsiveness can best be counteracted by modifying the dose of the physical exercise and/or physical training (Churchward-Venne et al., 2015; Ross et al., 2015; Montero and Lundby, 2017; Toigo, 2019). The latter suggests that the dose of physical interventions *per se* contributes significantly to the observed interindividual heterogeneity in (neurocognitive) outcomes.

### What Dose (It) Means?

The terminus dose is differently defined in the literature (Voils et al., 2012), but in exercise(-cognition) research, “dose” is commonly referred to as the product of exercise variables (e.g., exercise intensity, exercise duration, type of exercise; see Table 2) when considering an acute bout of physical exercises (Wasfy and Baggish, 2016; Pontifex et al., 2018). In training studies, dose can be seen as the product of exercise variables (e.g., exercise intensity, exercise duration, type of exercise), training variables (e.g., frequency of training sessions), and the application of training principles (Wasfy and Baggish, 2016; Northey et al., 2017; Solomon, 2018; Cabral et al., 2019; Erickson et al., 2019; Etnier et al., 2019; Falck et al., 2019; Ross et al., 2019; Williams et al., 2019). In reverse, dose could be modified in acute physical exercise studies by adjusting the exercise variables, while in physical training studies, exercise variables, training variables, and training principles must be taken into account (see Table 2). Such a purposeful modification is referred to as the adjustment of the exercise prescription.

**TABLE 2:** Overview of general exercise variables, training variables, and training principles.

**General exercise variables relevant in a single session**	
Exercise intensity	The exercise intensity describes how strenuous the exercise is.
Exercise duration	Time period that is spent for a specific exercise or the entire exercise session.
Type of exercise	Type(s) of exercise(s) that is (are) used in the exercise session (e.g., cycling, dancing).
**General training variables relevant in a training program**	
Frequency	The number of training sessions across a distinct time interval.
Density	Distribution of training sessions across a distinct time interval with regard to recovery time in-between training sessions.
Duration	Duration over which a training program is carried out.
**General training principles relevant in a training program**	
Variation	To prolong adaptations over a distinct training duration, systematic manipulation (variation) of exercise variables and training variables is necessary.
Specificity	To elicit a desired adaptation, the stimuli provided by the used physical exercises must be tailored to the desired adaptations (s).
Overload	To improve a distinct type of fitness, an appropriate stimulus must be provided that exceeds the already-existing individual capacities to a distinct extent.
Progression	To ensure continuous improvements, the stimulus must be appropriately modified over time (e.g., increase in external load).
Reversibility	Once the physical intervention induced stimulus is removed (e.g., stop the training), de-adaptational process will occur, and the changes in fitness level will eventually return to the baseline level.
Periodization and programming	In this context, periodization and programming are crucial elements for an appropriate exercise prescription. Periodization is the temporal coordination of training periods with specific fitness characteristics (e.g., strength or endurance) and application of training principles, which is referred to as macromanagement. Programming describes the organization of exercise variables and training variables (micromanagement). Periodization includes various forms such as linear periodization (LP) or non-linear periodization (NLP). In LP, typically, a gradual increase in intensity is conducted, whereas in NLP, exercise prescription is changed on weekly or daily basis.

**The definitions are based on Stone et al. (2002), Ratamess et al. (2009), Campbell et al. (2012), Winters-Stone et al. (2014) and Törpel et al. (2018).**

In the context of exercise prescription, it is also imperative to clarify the terms “external load” and “internal load.” While external load along with influencing factors (e.g., climatic conditions, equipment, ground condition) is defined as the work completed by the individual independent of internal characteristics (Wallace et al., 2009; Halson, 2014; Bourdon et al., 2017; Burgess, 2017; Vanrenterghem et al., 2017; McLaren et al., 2018; Impellizzeri et al., 2019), internal load is defined as individual and acute biomechanical, physiological, and/or psychological response(s) to the influencing factors and the work performed (Wallace et al., 2009; Halson, 2014; Bourdon et al., 2017; Burgess, 2017; Vanrenterghem et al., 2017; McLaren et al., 2018; Impellizzeri et al., 2019). According to the definition of internal load, which states that internal load is characterized by the individual and acute psychophysiological response(s) to the external load, it appears that internal load can be adjusted by modifying the external load. However, given that exercise variables such as exercise intensity can be operationalized using parameters of either external load (e.g., running with a speed of 10 km/h) or internal load (e.g., running with 60 of maximal heart rate), current definitions of dose are rather broad. Since dose is an essential factor for triggering neurobiological processes (e.g., release of neurotrophins such as BDNF; Dinoff et al., 2017), which in turn lead to neuroplastic and cognitive changes (Cotman et al., 2007; Voss et al., 2011, 2013a; Lucas et al., 2015; Zimmer et al., 2015; Basso and Suzuki, 2017; Stimpson et al., 2018), it is crucial to agree on an appropriate concept of dose. Although markers of internal load could be more difficult to measure (compared to markers of external load), we suggest that dose should be operationalized by using a specific marker or specific markers of internal load as a proxy. The two reasons for this assumption are outlined in the following.

#### Why Internal Load Should Be Used as a Proxy for Dose

Given (i) that internal load equals, per definition, the individual and acute psychophysiological response(s) to a given external load (Wallace et al., 2009; Halson, 2014; Bourdon et al., 2017; Burgess, 2017; Vanrenterghem et al., 2017; McLaren et al., 2018; Impellizzeri et al., 2019) and (ii) that neurocognitive changes are triggered by such distinct psychophysiological responses (Cotman et al., 2007; Zimmer et al., 2015; Basso and Suzuki, 2017; Stimpson et al., 2018), it seems reasonable to assume that internal load is a better proxy for dose than external load.

#### Why a Specific Marker of Internal Load Is Needed as a Proxy for Dose

There are several markers of internal load that can be used to prescribe the exercise intensity in acute endurance exercises and/or endurance training [e.g., oxygen uptake, heart rate, or heart rate variability (HRV)]. For instance, HRV, i.e., the beat-to-beat variation over a distinct time period, under rest conditions or while exercising is an interesting marker of internal load because the internal load quantification by HRV indices is progressive and takes the individual fitness level as well as daily readiness and actual health state into account (Thayer et al., 2012; Plews et al., 2013; Vesterinen et al., 2013, 2016; Gronwald et al., 2016, 2018a, 2019b,c). Furthermore, resting-state HRV is associated with cognitive performance (Hansen et al., 2003; Frewen et al., 2013; Gillie et al., 2014; Zeki Al Hazzouri et al., 2014; Colzato et al., 2018).

However, currently, several hypotheses exist in literature that explain the positive effects of acute physical exercises and physical training on brain plasticity and cognition (Kramer et al., 1999; Smiley-Oyen et al., 2008; Davenport et al., 2012; McMorris and Hale, 2015; McMorris, 2016a, b,c,d; McMorris et al., 2016; Voss, 2016; Raichlen and Alexander, 2017; Pontifex et al., 2018; Stimpson et al., 2018; Audiffren and André, 2019). Among them, one of the most popular hypotheses is the “neurotrophic hypothesis,” which posits that in response to physical exercises, the organism releases several neurochemicals (e.g., neurotrophic factors such as BDNF), which in turn trigger neuroplasticity and facilitate cognitive enhancement (Voss et al., 2013b; Basso and Suzuki, 2017; Stimpson et al., 2018; Audiffren and André, 2019). Hence, it seems more promising to use a marker or markers of internal load that are related to changes in neurotrophic molecules in order to individualize and adjust exercise prescription (Pedersen, 2019). In this regard, the peripheral level of blood lactate could be a promising marker of internal load because peripheral blood lactate (e.g., from muscles) can cross the blood–brain barrier and provides energy to the brain (Kemppainen et al., 2005; Quistorff et al., 2008; van Hall et al., 2009; Dennis et al., 2015; Proia et al., 2016; Taher et al., 2016; Riske et al., 2017; Brooks, 2018; Sobral-Monteiro-Junior et al., 2019). Hence, it is not surprising that relative changes in peripheral levels of blood lactate are correlated significantly with cognitive performance levels after high-intensity interval endurance exercises (Lee et al., 2014; Tsukamoto et al., 2016; Hashimoto et al., 2017). The crucial role of blood lactate for neuroplasticity is further emphasized by findings of peripheral blood lactate levels being associated with the peripheral serum BDNF levels (Ferris et al., 2007; Schiffer et al., 2011). However, the exact molecular mechanisms of increased BDNF production in response to physical exercising are not fully understood (for review, see Jiménez-Maldonado et al., 2018). BDNF in the brain is involved in neuroplasticity (Brigadski and Leßmann, 2014), and serum levels of BDNF have been shown to be directly linked to cognitive performance after an acute bout of high-intensity endurance exercises (Hwang et al., 2016). Moreover, (i) serum BDNF mediates improvements in cognitive functions following a 1-year aerobic endurance training (Leckie et al., 2014), (ii) greater serum BDNF concentration changes in response to a 1-year-long aerobic endurance training are linked to hippocampal volume changes (Erickson et al., 2011), and (iii) reduced levels of serum BDNF are related to a decline in hippocampal volume and poorer memory performance (Erickson et al., 2010). In sum, a specific marker or specific markers of internal load such as the peripheral blood lactate level seems to constitute a promising proxy for dose. However, the optimal marker(s) that is (are), with regard to neuroplasticity and cognition, the most suitable proxy for the dose of physical exercise and/or physical training has yet to be discovered.

### Become Personal – How to Individualize the Exercise Prescription?

**FIGURE 1:**
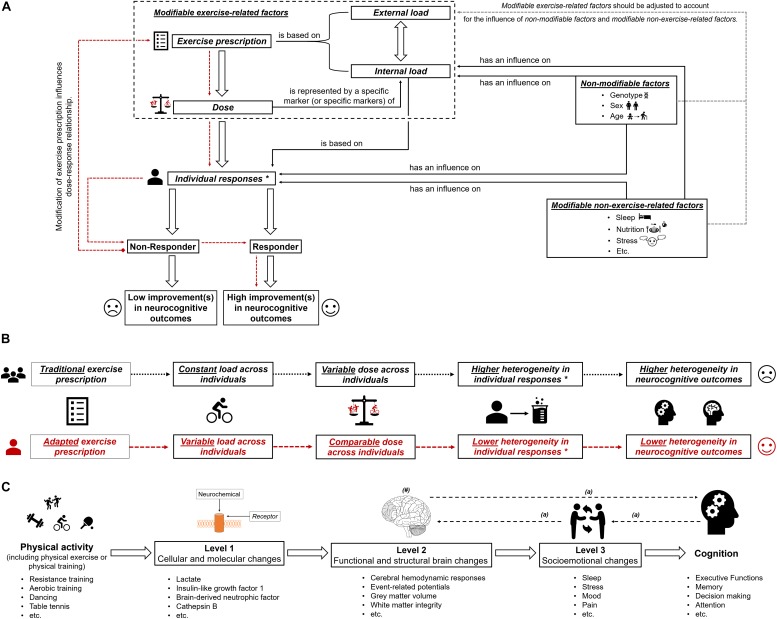
**(A)** Schematic illustration of the possible influence of exercise prescription on dose, and individual responsiveness (responder and non-responder) with the assumed extent of improvements (high improvements in neurocognitive outcomes and low improvements in neurocognitive outcomes). The dotted red lines show that by using an appropriate exercise prescription, non-responders could be turned into responders. In part **(B)** of the figure, the difference between “traditional exercise prescription” and “adapted exercise prescription” regarding the load, the dose, the individual response(s), and the corresponding heterogeneity in outcomes is illustrated. “^∗^” with regard to subsequent neurobiological processes. In part **(C)** of the figure, the multiple levels on which physical activity (including physical exercise and physical training) could affect cognitive performance are shown (Stillman et al., 2016). “#” indicates that the brain could be seen as outcome, mediator, or predictor (Stillman and Erickson, 2018). “a” indicates that there are several possibilities in which way structural and functional brain changes, socioemotional changes and cognitive changes are intertwined (Stillman et al., 2016).

Based on the large interindividual heterogeneity (i) in psychophysiological responses to acute physical exercises and (ii) in long-term adaptions to a physical training, it is assumed that tailoring of these to the characteristics and needs of a particular person is well suited to maximize their efficiency (Buford and Pahor, 2012; Buford et al., 2013; Müller et al., 2017, 2018; Cobbold, 2018; Pickering and Kiely, 2018b). Such an individualization of acute physical exercises and/or physical training could be achieved by adjusting the exercise prescription (e.g., exercise intensity) (Lightfoot, 2008), which influences, in turn, the dose (objectified by a specific marker or specific markers of internal load; see previous section and Figure 1A). In order to illustrate our thoughts in practical terms, we focus on exercise intensity because a full discussion of all exercise variables, training variables, and training principles is beyond the scope of this article. As outlined in the previous section, using markers of internal load to prescribe exercise intensity is preferable instead of using parameters of external load such as speed in running specific exercises. Therefore, traditional markers of internal load such as the fixed percentage of the maximally achievable value of oxygen uptake or heart rate are often used (Garber et al., 2011; Suwabe et al., 2018). Using a fixed percentage of a maximally achievable value of oxygen uptake or heart rate involves a considerable amount of interindividual heterogeneity in other markers of internal load (e.g., metabolic responses objectified by, for instance, peripheral blood lactate) (Weltman et al., 1989, 1990; Meyer et al., 1999; Vollaard et al., 2009; Scharhag-Rosenberger et al., 2010). Metabolic responses (e.g., peripheral blood lactate level) constitute specific markers of internal load that are likely to be proxies for the dose that triggers neuroplastic processes and cognitive changes (see “Why a specific marker of internal load is needed as a proxy for dose”). Hence, traditional exercise prescriptions lead to largely varying individual doses as revealed by the marker(s) of internal load. This may lead, among other factors, to the observed interindividual heterogeneity in neurocognitive outcomes (see Figure 1B). Consequently, approaches that ensure that a comparable dose is provided to each individual (e.g., adapted exercise prescriptions that ensure a comparable level of peripheral blood lactate) may lower the interindividual heterogeneity regarding neurocognitive outcomes. Hence, such approaches are favorable in exercise–cognition research (see Figure 1). In this context, individual threshold concepts (aerobic and anaerobic threshold) that are based on individual metabolic (or respiratory) responses could be used to determine an individual’s initial exercise intensity (Meyer et al., 1999; Hofmann and Tschakert, 2010; Scharhag-Rosenberger et al., 2010; Weatherwax et al., 2016). However, while there is a strong theoretical basis for the application of a threshold-based exercise prescription for endurance exercises and endurance training, the challenges and pitfalls of determining such individual thresholds may explain why many researchers continue to favor exercise intensity prescriptions based on relative percentages of maximum values (Hofmann and Tschakert, 2010; Mann et al., 2013). Although our assumptions are well grounded on possible neurobiological mechanisms, they are mostly theoretical in nature, and thus, further research comparing, for instance, traditional versus adapted exercise prescriptions with regard to neuroplasticity and cognition is urgently needed.

### Progress Is Not Without Limitations

Since the level and detail of description required to extensively describe and discuss the influence of all exercise variables, training variables, training principles, and factors influencing exercise–cognition interaction go far beyond the scope and intent of this article, our assumptions still remain imperfect because other exercise-related factors such as movement frequency (e.g., cycling cadence) (Ludyga et al., 2015, 2016) or psychological factors such as affective response (e.g., enjoyment or expectations) (Davidson et al., 2000; Davidson and McEwen, 2012; Burnet et al., 2018; Lindheimer et al., 2019) have not been considered. Nevertheless, given (i) that our knowledge of the dose–response relationship between acute physical exercises and/or physical training, neurobiological processes (e.g., neuroplasticity), and cognitive changes is still limited (Etnier et al., 2006, 2019; Hillman et al., 2008; Chang et al., 2012a, b; Barha et al., 2017b; Ströhlein et al., 2017; Tait et al., 2017; Pontifex et al., 2018; Stimpson et al., 2018; Erickson et al., 2019; Falck et al., 2019; Sanders et al., 2019), (ii) that peripheral blood lactate levels constitute an established marker of internal load (Hofmann and Tschakert, 2010; Beneke et al., 2011; Soligard et al., 2016; Impellizzeri et al., 2019), and (iii) that peripheral blood lactate levels are easily quantifiable by portable devices, the use of peripheral blood lactate as a proxy for dose seems a reasonable starting point. Nevertheless, lactate monitoring suffers from the drawbacks that (i) it necessitates blood sampling, which could be impractical in daily practice, and (ii) it requires a graded exercise test to calculate an individual threshold to prescribe the exercise intensity. Regarding the first objection, new methods to non-invasively determine critical physiological thresholds (e.g., lactate threshold) by means of muscle near-infrared spectroscopy (Wang et al., 2006; Xu et al., 2011; Bellotti et al., 2013; Borges and Driller, 2016; Driller et al., 2016) may constitute a more appropriate approach in daily practice, but this has yet to be investigated. With regard to the second objection, it is worth mentioning that graded exercise tests are relatively complex and time consuming and that exercise intensity could be more easily determined by using specific formulas (e.g., Karvonen formula to determine a target heart rate) (Karvonen and Vuorimaa, 1988; Tanaka et al., 2001; Gellish et al., 2007; Zhu et al., 2010; Nes et al., 2013; Shargal et al., 2015). However, a graded exercise test should be an integral part of the process of a proper exercise prescription because, currently, exercise intensity cannot be accurately predicted by specific formulas (Strzelczyk et al., 2001; Robergs and Landwehr, 2002; Silva et al., 2007; Sarzynski et al., 2013; Correa Mesa et al., 2015; Esco et al., 2015; Arena et al., 2016), and a fixed percentage of a maximally achievable value of heart rate leads to a considerable amount of interindividual heterogeneity in metabolic responses (e.g., blood lactate) (Meyer et al., 1999), which is deemed to contribute, at least partly, to the interindividual heterogeneity in neurocognitive outcomes (see previous sections).

Still, even if peripheral blood lactate concentrations are associated with serum BDNF concentrations (Ferris et al., 2007; Schiffer et al., 2011), further studies will be required to investigate the dose–response relationship between exercise prescription and (serum) BDNF levels (Knaepen et al., 2010; Coelho et al., 2013; Huang et al., 2014). Since BDNF release is also influenced by several other non-modifiable (e.g., sex Trajkovska et al., 2007; Komulainen et al., 2010; Bus et al., 2011) or non-exercise-related modifiable factors (e.g., sleep or nutrition; Giese et al., 2013, 2014; Walsh et al., 2015; Schmitt et al., 2016) that are known to influence neuroplasticity in general (e.g., sleep, Meerlo et al., 2009; Raven et al., 2018; or nutrition, Greenwood and Parasuraman, 2010; Phillips, 2017; Poulose et al., 2017), these factors should be carefully monitored in further studies.

In addition, with regard to the optimal dose, it could be useful to gather markers of internal load that are directly related to the state of the central nervous system itself (e.g., brain activity during exercise) because differences in brain activity (e.g., measured by functional near-infrared spectroscopy) (i) allow distinguishing between responders and non-responders (Yamazaki et al., 2017), (ii) are sensitive to changes of exercise variables (e.g., exercise intensity) (Rooks et al., 2010; Giles et al., 2014; Tempest et al., 2014; Santos-Concejero et al., 2015, 2017; Takehara et al., 2017), (iii) are sensitive to demands posed by the cognitive task (Herff et al., 2013; Fishburn et al., 2014; Causse et al., 2017; Khaksari et al., 2019) or the motor task (Carius et al., 2016), (iv) and are associated with performance improvements in motor(–cognitive) tasks (Ono et al., 2014, 2015; Herold et al., 2017; Seidel et al., 2017). Hence, markers of internal load assessing activation of the central nervous system may serve to quantify “complexity” (defined as neurocognitive demands posed by the exercise), which is an important variable with regard to neurocognitive changes in response to acute physical exercises and physical training, too (Netz, 2019). However, while measuring brain activation during exercise offers great potential to understand exercise–cognition interaction in general and interindividual variability in particular, future research in this area is strongly needed before measures of brain activity can be used to guide exercise prescription.

Furthermore, we wish to stress that a traditional individualization of exercise prescription is perhaps necessary to answer basic research questions (e.g., *Are the peripheral blood lactate release and changes in neurocognition a function of exercise intensity?*) but that the individualization using an adapted exercise prescription may lead to further insights into exercise–cognition research (e.g., *How to adapt exercise intensity to achieve a comparable change in the release of peripheral blood lactate across individuals and how this affects neurocognition?*).

## Conclusion and Further Remarks

In essence, this article aimed at providing a suggestion for a clearer definition of the dose in exercise–cognition research and presenting evidence in how interindividual variability in the dose might contribute to the interindividual heterogeneity in neurocognitive outcomes. We propose that the dose of an acute bout of physical exercises and/or physical training should be operationalized by a specific marker (or specific markers) of internal load. Modifying the exercise prescription by carefully adjusting the external load, a comparable dose can be achieved across individuals (see Figures 1A,B). Research is strongly encouraged to investigate in the future whether an exercise prescription inducing a comparable dose may lower the interindividual heterogeneity considering outcome variables on different levels of analysis (Stillman et al., 2016) and on different aspects of the brain (Stillman and Erickson, 2018; see Figure 1C). Finally, understanding how a comparable dose affects neurocognitive outcomes is an important step toward identifying what dose is optimal for achieving the greatest benefits with regard to neurocognitive outcomes in an individual.

## Author Contributions

FH wrote and edited the manuscript. PM, TG, and NM reviewed and edited the drafted versions.

## Conflict of Interest

The authors declare that the research was conducted in the absence of any commercial or financial relationships that could be construed as a potential conflict of interest.
